# Principles for interactions with biopharmaceutical companies: the development of guidelines for patient advocacy organizations in the field of rare diseases

**DOI:** 10.1186/s13023-018-0761-2

**Published:** 2018-01-22

**Authors:** Susan Stein, Elizabeth Bogard, Nicole Boice, Vivian Fernandez, Tessa Field, Alan Gilstrap, Susan R. Kahn, Jane Larkindale, Toni Mathieson

**Affiliations:** 1 Connexion Healthcare, 6 Terry Drive, Newtown, PA 18940 USA; 2 International Fibrodysplasia Ossificans Progressiva Association (IFOPA), 1520 Clay Street, Suite H-2, North Kansas City, MO 64116 USA; 3 Global Genes, 28 Argonaut, Suite 150, Aliso Viejo, CA 92656 USA; 4 REGENXBIO Inc, 9600 Blackwell Road, Suite 210, Rockville, MD 20850 USA; 5 Spark Therapeutics, 230 Third Avenue, 6th Floor, Waltham, MA 02451 USA; 6 Akcea Therapeutics, 55 Cambridge Parkway, Cambridge, MA 02142 USA; 7 National Tay-Sachs & Allied Diseases Associations Inc, 2001 Beacon Street, #204, Brighton, MA 02135 USA; 8 Friedreich’s Ataxia Research Alliance, 6130 N Canon del Pajaro, Tucson, AZ 85750 USA; 9 Niemann-Pick UK, Suite 2, Vermont House, Concord, Washington, Tyne and Wear, NE37 2SQ UK

**Keywords:** Best practices, Biopharmaceutical, Guidelines, Partnership, Patient advocacy, Rare disease, Standards

## Abstract

**Background:**

Rare diseases are a global public health concern, affecting an estimated 350 million individuals. Only 5% of approximately 7000 known rare diseases have a treatment, and only about half have a patient advocacy organization. Biopharmaceutical companies face complex challenges in developing treatments for rare diseases. Patient advocacy organizations may play a major role by positively influencing research and development, clinical trials, and regulations. Thus, collaboration among patient advocacy organizations and industry is essential to bring new therapeutics to patients.

**Methods:**

We identified an unmet need for guidelines on day-to-day decision-making by rare disease patient advocacy organizations when working with biopharmaceutical partners. We convened an Independent Expert Panel experienced in collaborations between patient advocacy organizations and biopharmaceutical companies (April 2017) to develop consensus guidelines for these relationships. The guidelines were based on an original version by the International Fibrodysplasia Ossificans Progressiva Association (IFOPA). The Expert Panel reviewed and broadened these to be applicable to all patient advocacy organizations. Comments on the draft Guidelines were provided first by Panel participants and subsequently by six independent experts from patient advocacy organizations and industry.

**Results:**

The Panel comprised four experts from the rare disease community who lead patient advocacy organizations; three leaders who perform advocacy functions within biopharmaceutical companies; and two facilitators, both having leadership experience in rare diseases and industry. The finalized Guidelines consist of four main sections: Identification and Engagement With Companies, Patient Engagement and Patient Privacy, Financial Contributions, and Clinical Trial Communication and Support. The Guidelines address the daily considerations, choices, and consequences of patient advocacy organizations as they engage with biopharmaceutical companies, and offer recommendations for volunteer/paid leaders of the organizations on how to interact in a thoughtful, responsible, ethical way that engenders trust.

**Conclusions:**

These Guidelines recommend best practices and standards for interactions between patient advocacy organizations and industry that will ultimately have a positive effect on the development of novel treatments. Patient advocacy organizations will be provided free access to these Guidelines to help bring clarification to day-to-day decision-making around their interactions, and for use as a living document with the potential for regular revisions and updates.

## Background

Rare diseases are a global public health concern, affecting an estimated 350 million individuals. The definition of a rare disease varies internationally [1]. In the United States, a disease is considered rare if it affects fewer than 200,000 individuals at any given time; in the United Kingdom, rare disease is defined as a disease that affects fewer than 50,000 individuals. Although the number of patients with each specific rare disease is small, collectively, rare diseases affect about 30 million people in the United States and another 30 million in Europe [1].

In the United States, the Orphan Drug Act was designed to provide incentives to manufacturers to conduct research and development for rare disease therapies [2]. Although the submission and approval rates for orphan drugs in the United States have increased since the passage of the Act in 1983 [2, 3], only 5% of the approximately 7000 rare diseases identified currently have a treatment approved by the US Food and Drug Administration (FDA) [1].

Rare diseases are confronted with some of the most complicated scientific and medical challenges of today. There are numerous barriers to the development of new and meaningful treatments, including unanswered scientific questions; a lack of data on disease epidemiology, pathophysiology, and natural history; a lack of clearly defined biomarkers to measure disease activity; and difficulty in identifying clinical trial endpoints and measurable and validated disease outcomes [1, 4, 5]. The technical challenges are compounded by limited research funding for rare diseases compared with that provided for more common diseases, and by difficulty in finding and recruiting patients to participate in clinical studies [3]. Finally, patients with rare diseases and academic experts are often geographically dispersed, making it more difficult for drug developers to locate and engage with them. Expertise in the care and management of a particular rare disease is often concentrated in a small number of academic centers that may not be experienced in clinical research or development, adding to the time and costs of starting and implementing clinical trials. Patient advocacy associations are a key interface between patients and providers.

Solving the challenges posed by rare diseases requires the collaboration of multiple stakeholders: biopharmaceutical companies, academic researchers, clinicians, patient advocacy organizations, patients, and regulators. Yet collaboration, while essential, is often challenging. The highest personal, professional, and business stakes are at play; considerable ethical and legal issues exist; incentives can be misaligned; and the environment is both incredibly complex and rapidly evolving.

Patient organizations are playing an increasingly important role in addressing and overcoming the challenges of drug development. About half of all known rare diseases are represented by a disease-specific patient advocacy organization [1]. This number is growing. Although the history of patient advocacy organizations lies in grass roots efforts and family fundraising events, the sophistication of the patient community has increased to match its ambition to find effective therapeutics. Many patient advocacy organizations today are led by professionals, are meaningful and empowered stakeholders in the development of treatments, and have a large impact on research [5–7].

Indeed, today, one of the most important factors in the development of new therapies is the positive influence of patient advocacy organizations on research and development, clinical trials, and governmental regulations [7].

Patient advocacy organizations perform many functions that enable drug development and access, including the following [5–7]:

- Educating patients, physicians, and the community about a disease and innovations in its management and treatment
- Pushing the pace of research by championing and directly funding efforts to increase the understanding of the cause of a disease and to develop new therapies
- Forming connections between disease experts and drug developers
- Providing drug developers with relevant insights into the patient community to enable the development of therapies that best meet the community’s needs
- Advocating for and influencing changes in regulations to expedite research
- Facilitating and/or sponsoring patient registries and natural history studies to aid in the development of treatments
- Helping to ensure that patients have access to treatments

With the evolution of patient advocacy organizations and the increasing activity of biopharmaceutical companies in the development of novel therapies for rare diseases, collaborations between patient advocacy organizations and biopharmaceutical companies have become more common. However, many organizations face uncertainty in addressing the complex and important day-to-day issues that arise within the context of these collaborations.

Although guidance documents exist, most either are directed at the biopharmaceutical industry, are specific to the European healthcare system [8, 9], or are too general to serve as a roadmap for day-to-day decision-making for patient advocacy organizations. Given the important and dynamic nature of their work and the complexity of collaboration in the development of treatments for rare diseases, many patient advocacy organizations are seeking clarity and guidance concerning approaches to industry collaboration.

Guidelines are needed now more than ever, especially with healthcare regulatory bodies increasingly restructuring themselves to put the needs of patients first. The European Medicines Agency (EMA) established the Patients’ and Consumers’ Working Party (PCWP) in 2006 to provide a platform for information exchange between patients and the Agency. The US FDA enacted the US twenty-first Century Cures Act in December 2016 [10]. The Act seeks to make improvements throughout the entire research and development system, from discovery, to development, to delivery of new medical products [10]. The policy reforms in the Act are meant to strengthen patient centricity in the areas of biomedical product development and regulatory approval and to catalyze innovation in clinical trials and regulatory approval. Such innovations would include promoting FDA qualification of biomarkers and other drug development tools and the study of how best to use innovative clinical trial designs and the real-world evidence that is generated during product development and regulatory approval [10, 11]. In 2016, the EMA and the FDA established a new ‘cluster’ on patient engagement. This cluster is intended to provide a forum to share experiences and best practices on the way the two agencies involve patients in development, evaluation, and post-authorization activities related to medicines [12].

The great need for guidelines was most recently emphasized by the publication of an article in March 2017 in *The New England Journal of Medicine* addressing conflicts of interest that may arise when patient advocacy organizations interact with industry [13].

In order to address the need for guidelines, we convened an Independent Expert Panel of leaders from patient advocacy organizations and the biopharmaceutical industry to develop guidelines on effective collaborations between these two stakeholders. This report presents the objectives and guiding principles developed by the Independent Expert Panel, the process used to develop the guidelines, a summary of points raised during the Panel discussion, and the finalized guidelines.

### Objectives

The objectives of the Independent Expert Panel meeting, titled “Principles for Interactions With Pharmaceutical Companies: Guidelines for Patient Organizations,” were as follows:

- To create guidelines directed at day-to-day decision-making for rare disease patient advocacy organizations for use in working with industry partners
- To capture comments and suggestions from participants for use in developing a white paper on the principles of interactions with industry to support the guidelines
- To determine next steps regarding finalization and dissemination of the manuscript and the guidelines

## Methods

Nine experts chosen for their expertise and availability participated in the Independent Expert Panel: four leaders of patient advocacy organizations and three leaders in the pharmaceutical industry who interact with patient advocacy organizations. The Panel also included two facilitators, one with experience in such collaborations at both a patient advocacy organization and a biopharmaceutical company (EB) and one from a medical communications company specializing in rare diseases (SS).

The Independent Expert Panel meeting utilized a roundtable discussion format. The meeting was held via a 2-h web conference, ensuring that each participant was given the opportunity to provide input into the guidelines. The agenda included background and introductions, questions for discussion, a session on other comments and suggestions, and next steps.

Prior to the meeting, the Panel was provided with and asked to review the known guidance documents available on this topic **(**Table 1**)** [13–17]. The Panel was also provided with a set of guidelines previously developed by the International Fibrodysplasia Ossificans Progressiva Association (IFOPA), a nonprofit patient advocacy organization for this rare disease founded in 19889 (see www.ifopa.org), titled “The International Fibrodysplasia Ossificans Progressiva Association’s (IFOPA) Guidelines for Engagement with Pharmaceutical Companies” [18].

**Table 1:** Background materials for the Independent Expert Panel meeting

Existing Guidance Documents	
• Pharmaceutical Manufacturers of America (PhRMA) (United States): “PhRMA Principles on Interactions with Patient Organizations” [14].	
• Clinical Trials Transformation Initiative CTTI Recommendations (United States): “Effective Engagement with Patient Groups Around Clinical Trials” [15].	
• European Federation of Pharmaceutical Industries and Associations (Europe): “EFPIA Code of Practice on Relationships Between Pharmaceutical Companies and Patient Organisations” [16].	
• BioPontis Alliance for Rare Diseases (United States and Europe): “Integrating Rare Disease Patients into Pre-Clinical Therapy Development; Finding Our Way with Patient Input” [17].	
Published Literature	
• McCoy MS, Carnoil M, Chockley K, Urwin JW, Emanuel EJ, Schmidt H: Conflicts of interest for patient-advocacy organizations. *N Engl J Med* 2017;376:880–5.	

The IFOPA guidance document provided a foundation from which the Panel was asked to build a set of guidelines for use by the entire rare disease community. The IFOPA document was chosen because it reflects many of the broader principles described by the known guidance documents listed in Table 1. The facilitators discussed and raised key questions for each of the five sections of the IFOPA guidance document **(**Table 2**)** [18]:

1. Identification and Engagement of Companies
2. Patient Engagement
3. Financial Contributions
4. Clinical Trial Communications
5. Patient Privacy

**Table 2:** Discussion questions posed to the participants of the Independent Expert Panel meeting^a^

1. Identification and engagement of companies	
1.1. Are there any conditions under which a patient advocacy organization should not be in contact with a pharmaceutical company?	
1.2. Are there criteria a patient advocacy organization should use to decide whether or which companies to contact?	
1.3. What information should a patient advocacy organization expect a company to share?	
1.4. What information should a company expect a patient advocacy organization to share?	
2. Patient engagement	
2.1. Should a patient advocacy organization be involved in direct interaction between a patient and a pharmaceutical company? What does “involvement” in this case mean?	
2.2. Are there circumstances under which a patient advocacy organization should *not* be involved in direct dialogue between patients and companies?	
2.3. What are the reasons behind this recommendation?	
2.4. For formal disease insight, is an advisory board preferred over individual input? Are there circumstances under which individual input is preferred or acceptable?	
3. Financial contributions	
3.1. Should patient groups accept financial contributions from pharmaceutical companies? If so, under what circumstances?	
3.2. By what mechanisms should a patient group receive contributions? What processes should be in place for receiving and reporting the contribution?	
3.3. Should patient group leaders accept honoraria for speaking on behalf of their organization?	
3.4. Are there circumstances under which a patient advocacy organization could/should operate as a paid service provider to a pharmaceutical company?	
4. Clinical trial communications	
4.1. What is the role of a patient advocacy organization in “supporting” clinical trials?	
4.2. Should a patient advocacy organization have criteria for which clinical trials it will “support”?	
4.3. What role, if any, should a patient advocacy organization have in guiding or advising conversations about clinical trial participation on social media?	
4.4. Should leaders of patient advocacy organizations (board members and other volunteer leaders) follow organizational practices when writing or speaking in personal blogs or other social media as a parent, family member, or affected individual?	
5. Patient privacy	
5.1. What role does a patient advocacy organization have in ensuring that pharmaceutical companies adequately protect patient privacy in surveys, advisory boards, or other community engagement?	

^a^ The questions listed herein apply to sections 1 through 5 of the Guidelines for Engagement with Pharmaceutical Companies by The International Fibrodysplasia Ossificans Progressiva Association (IFOPA) [18]

During the meeting, the Panel addressed the key questions (Table 2) and provided comments and revisions to the IFOPA document. Based on input from the Panel, the authors developed a first draft guidance document.

## Results

The Independent Expert Panel reviewed and revised this first draft. A second draft was also reviewed by six independent experts from patient advocacy organizations and the biopharmaceutical industry. The Guidelines were finalized in October 2017.

The Guidelines produced by the Independent Expert Panel, titled “Guidelines for Interactions Between Patient Advocacy Organizations and Biopharmaceutical Companies,” are provided in the **Appendix** to this article. The Panel adapted the original five sections of the IFOPA guidelines into four sections:

1. Identification and Engagement With Companies
2. Patient Engagement and Patient Privacy
3. Financial Contributions
4. Clinical Trial Communication and Support

The Guidelines address the day-to-day considerations, choices, and consequences for patient advocacy organizations in their engagement with biopharmaceutical companies. The Panel agreed that all interactions between the patient advocacy organization, industry, and the disease community should be transparent; should enable trust, accountability, and shared learning; and ultimately should work most efficiently and effectively toward advancing meaningful treatments for patients. The Guidelines strive to achieve these objectives while recognizing that every relationship is unique and that there are a variety of ways to partner successfully.

The recommendations contained within the Guidelines are relevant for any paid or volunteer leader of a patient advocacy organization, including staff, board members, and committee members. The Guidelines are intended to be a living resource that can be revised and expanded as the environment evolves.

Participants in the Expert Panel stressed the importance of having the Guidelines available and published in the literature. The Panel also provided the following overarching comments:

- Patients and industry cannot “go it alone.” Both need patient advocacy organizations.
- Patient advocacy organizations may not realize their value and how important they are to industry.
- Successful collaborations between patient advocacy organizations and biopharmaceutical companies are achieved when they have reciprocal relationships in which both parties recognize the value of the other.
- Mutual respect is essential, which requires honesty and authenticity. Transparency and commitment from both parties should begin on day one.

The Independent Expert Panel provided Probing Questions for possible use in conjunction with the Guidelines **(**Table 3**)**. The Panel also recommended that patient advocacy organizations develop educational resources for patients **(**Table 4**)**.

**Table 3:** Probing questions from the Independent Expert Panel meeting, for possible use with the Guidelines

Identification and Engagement With Companies	
• What changes occur when more than one patient advocacy organization is involved?	
• What changes occur when more than one industry partner is active?	
• How can the challenges of having only one biopharmaceutical company in the particular rare disease space be addressed?	
Patient Engagement and Patient Privacy	
• What does a great patient advocacy organization look like?	
• What are the ways that individual patient advocacy organizations can facilitate bringing back information to the disease community?	
• What are some reasons that patient advocacy organizations should be included in conversations with industry?	
• How should patient advocacy organizations handle various aspects of confidential information as an organization?	
Financial Contributions	
• Should there be a cap on the total percentage of funding from pharmaceutical companies? Should the cap depend on the size of the group?	
• How should public disclosures of pharmaceutical funding be handled?	
• Can industry employees serve on the boards of patient advocacy organizations?	
Clinical Trial Communication and Support	
• How should patient advocacy organizations respond to misinformation on the Internet and in social media?	
• What changes occur once a drug is commercially available?	
• How should negative results of clinical trials be handled? How should that information be disseminated to the patient community?	
• How should patient advocacy organizations handle issues surrounding compassionate use?	
• Should patient advocacy organizations attempt to educate academic researchers on how to communicate with patients?	
• Should patient advocacy organizations provide education and resources to patients about the informed consent process?

**Table 4:** Education and resources that patient advocacy organizations can provide and share

1. Provide training on these Guidelines.	
2. Develop case studies of how patient advocacy organizations have worked with the biopharmaceutical industry in the past.	
3. Trade patient-friendly toolkits with other patient advocacy organizations.	
4. Provide education and guidance to patients concerning:	
○ Clinical trial participation; informed consent and how to make a decision about participating in a clinical trial	
○ The use of social media around clinical trials	
○ How patients should interact with industry regarding participation in advisory groups	
5. Provide education for academic researchers about how to communicate information about clinical trials with patients.	

## Discussion

Collaborations between patient advocacy organizations and the biopharmaceutical industry, which have become increasingly more common, are needed now more than ever to overcome the challenges of developing treatments for rare diseases. The increasing focus on patient engagement by healthcare regulators highlights the need for guidelines to facilitate this collaboration. Although collaboration is essential, it is also poses challenges. The Independent Expert Panel developed Guidelines that are intended to help address these challenges. The Guidelines presented here are directed at day-to-day decision-making for rare disease patient advocacy organizations for use in working with industry partners. Comments and suggestions from the members of the Independent Expert Panel were included in the Guidelines, and the Expert Panel reviewed and finalized the Guidelines. Additional comments on the draft Guidelines were provided by independent experts from patient advocacy groups and the biopharmaceutical industry.

Various limitations to the development of these Guidelines exist: the complexity of collaborations between patient advocacy groups and the biopharmaceutical industry, the dynamic nature of the work of patient advocacy organizations in the development of therapies for rare diseases, and various regional differences such as those between the United States and Europe related to interactions between these types of groups. However, these Guidelines are intended to be freely available for use as a living document, with the potential for regular revisions and updates. Future versions of the guidelines should explore regional differences and needs in how patient organizations interact with pharmaceutical companies, particularly between the United States and Europe.

## Conclusions

The Guidelines described here are intended to guide collaborations between patient advocacy organizations and the biopharmaceutical industry in an ethical and transparent way for the benefit of patients who desperately need novel and meaningful therapeutics. The Guidelines recommend best practices and standards for interactions between patient advocacy organizations and the biopharmaceutical industry that will ultimately have a positive effect on the development of novel treatments.

## Background

The interactions between patient advocacy organizations and biopharmaceutical companies are important and complex. Collaborations between these two stakeholders have become more common in recent years as patient advocacy organizations have evolved and biopharmaceutical industry activity has increased, particularly in rare diseases. Given the important and dynamic nature of their work and the complexity of drug development, many patient advocacy organizations desire clarity and guidance on effective approaches to engaging with the biopharmaceutical industry to realize their vision of meaningful, novel therapeutics.

The principles outlined in the following Guidelines are intended to help rare disease patient advocacy organizations navigate critically important interactions with biopharmaceutical companies. These Guidelines may be adopted in whole or modified to suit organizational needs and interests. There are many ways to partner. These Guidelines offer the optimal approach intended to best serve patient needs while recognizing that the complexity of drug development for serious health conditions warrants varied solutions.

These Guidelines were developed with input from an Independent Expert Panel from the rare disease community with expertise in these collaborations from either the industry or patient advocacy organization point of view. More information on the Independent Expert Panel and the process for developing these Guidelines is provided in the article entitled “Principles for Interactions With Biopharmaceutical Companies: The Development of Guidelines for Patient Advocacy Organizations,” published in The *Orphanet Journal of Rare Diseases,* 2018.

## Introduction

The patient advocacy organization seeks the highest level of ethical conduct in engagement with biopharmaceutical companies. The goal of engaging with biopharmaceutical companies is to help enable the development of therapies while maintaining autonomy as a patient advocacy organization. All interactions between the patient advocacy organization, industry, and the disease community should be transparent; should enable trust, accountability, and shared learning; and ultimately should work most efficiently and effectively toward advancing meaningful treatments for patients.

There are four main areas of engagement between the patient advocacy organization and biopharmaceutical companies described in the following Guidelines:

1. Identification and Engagement With Companies
2. Patient Engagement and Patient Privacy
3. Financial Contributions
4. Clinical Trial Communication and Support

**1. Identification and Engagement With Companies**

The patient advocacy organization desires mutually beneficial dialogue and information exchange with biopharmaceutical companies developing potential therapies for the rare disease of interest. Dialogue is mutually beneficial when it is aligned with and advances the mission of the patient advocacy organization as well as the biopharmaceutical company.

1.1. The patient advocacy organization proactively seeks contact with biopharmaceutical companies that show interest or activity in drug discovery, preclinical research, or clinical research in the rare disease of interest. The patient advocacy organization may also contact companies that are not yet working on the rare disease of interest but have a relevant technology.

1.2. The patient advocacy organization seeks insights into the objectives and plans of the biopharmaceutical company and the potential therapy being evaluated, as appropriate. In exchange, the patient advocacy organization provides the biopharmaceutical company with community-wide insight and perspective as needed and appropriate to inform the development efforts and strategic decisions of the company.

1.3. The patient advocacy organization collaborates with biopharmaceutical companies that are conducting ethical, high-quality research in a responsible manner according to industry, national, and international regulatory standards. Collaboration can include a wide range of activities such as information exchange, access to disease experts, access to tools and infrastructure (e.g., natural history data and biologic samples), and exchange of resources.

1.4. The patient advocacy organization strives to collaborate with multiple biopharmaceutical companies to ensure the sustainability of its initiatives and to allow for a diversity of views and therapeutic approaches.

1.5. The patient advocacy organization discusses goals and expectations of the collaboration at the outset to ensure they are mutual. The patient advocacy organization reserves the right to disengage with a biopharmaceutical company if the goals of the two organizations are not aligned.

1.6. In order to avoid conflicts of interest, the patient advocacy organization does not allow representatives of biopharmaceutical companies actively developing or selling therapies for the disease to sit on the board of directors of the patient advocacy organization.

**2. Patient Engagement and Patient Privacy**

The patient advocacy organization encourages and enables direct dialogue and information exchange between patients and biopharmaceutical companies developing potential therapies for the rare disease of interest. The voice of the patient is crucial throughout drug development by promoting disease awareness and sharing patient/caregiver perspectives. The patient advocacy organization ensures the privacy of health data provided to the organization by its membership.

2.1. Any engagement between a biopharmaceutical company and a patient advocacy organization should be done to advance understanding of the disease or research efforts and should have a clearly stated purpose or set of objectives.

2.2. Direct interactions between specific patients and biopharmaceutical companies are best arranged with the involvement or general awareness of the patient advocacy organization. The patient advocacy organization can choose from a range of approaches regarding these interactions, from actively facilitating such dialogues to passively providing training and education for patient community members on best practices.

Including the patient advocacy organization in these dialogues accomplishes the following:

- Ensures fairness and transparency within the patient community. Information provided by the biopharmaceutical company to one patient is shared with all patients who have a right to that information, and patients outside of the conversation have an equal opportunity to express their opinions to the biopharmaceutical company.
- Ensures that the patient community is adequately and well represented to the biopharmaceutical company. The voices of individual patients must be considered in the context of the community as a whole; one patient’s experience may not reflect the experiences of other patients.
- Allows for access to professional advisers, such as financial experts and attorneys, who may advise the organization, inform the dialogue, and help individual patients avoid financial and legal risks.
- Helps to avoid misunderstandings in conversations between individual patients and biopharmaceutical companies.
- Allows the organization to advise patients on the protection of their health and personal privacy in any data collection activities.
- Allows the patient advocacy organization to better understand the needs and intentions of both the patient and the biopharmaceutical company in order to best move the field forward for the patient community as a whole.

2.3. The patient advocacy organization encourages biopharmaceutical companies to obtain disease insights from group discussion rather than from one-on-one conversation with single individuals. One best practice is the formation of advisory boards composed of at least three patients. An advisory board format helps to ensure that community views are adequately represented and that work is not unduly requested of any one individual. The patient advocacy organization may offer guidelines and training to patients and families on best practices for effective interactions with biopharmaceutical companies as part of an advisory board.

2.4. The patient advocacy organization expects that learnings and outcomes from all interactions will be shared openly between both parties for mutual benefit. At the outset of these collaborations, the patient advocacy organization may offer the companies guidelines or expectations for how learnings and outcomes can best be shared with their particular community.

2.5. Leaders of the patient advocacy organization (i.e., staff, board members, and committee members) or individuals representing the disease community may be invited by biopharmaceutical companies to speak at internal company meetings, public events hosted by the company, or meetings with regulatory agencies. The patient advocacy organization evaluates each invitation and accepts invitations that promote disease education or awareness and elevate the voice of the patient in a manner that is consistent with the points outlined in these Guidelines.

2.6. The patient advocacy organization takes proper steps to protect all personal and confidential patient information both within the organization and when shared with outside entities, in accordance with applicable laws and regulations. The patient advocacy organization undertakes the following:

- Assists individual patients in thinking through their decisions about providing information or consent.
- Helps patients convey their expectations about privacy.
- Ensures that biopharmaceutical companies, and other organizations, have in place at least basic guidelines or a policy for ensuring patient privacy prior to any data collection, including surveys, photographs, video and audio recordings, slide decks, and consent forms.

2.7. The patient advocacy organization advises patients and industry that personal health information of patients must not be recorded by the biopharmaceutical company without proper and prior informed consent from the patient.

2.8. The patient advocacy organization advises patients and industry on the value of sharing data with the research community for future research needs, and encourages use of consent documents that allow for secondary research on data as permitted by patients. Patient advocacy groups may encourage sharing of data on completion of studies.

**3. Financial Contributions**

A robust patient advocacy organization is a vital partner to biopharmaceutical companies in the development of potential therapies. Financial resources are a key need for the growth and maintenance of the patient advocacy organization. Demands on the organization are increased by drug development activities, particularly during the clinical and commercial stages. The following principles guide the patient advocacy organization in the receipt of biopharmaceutical company donations:

3.1. The patient advocacy organization requires and maintains proper documentation of all requests for financial support from a biopharmaceutical company. All requests are documented on the letterhead of the organization and clearly state the mission and activities of the advocacy organization and reasons for the request.

3.2. The patient advocacy organization accepts financial contributions that support its stated mission and allow the organization to maintain its autonomy. The patient advocacy organization assesses the alignment of mission between the two organizations as part of the funding discussion.

3.3. The patient advocacy organization does not accept financial support from biopharmaceutical companies for product promotional activities. The patient advocacy organization avoids taking payment from a biopharmaceutical company that could be perceived as buying special privileges, such as the opportunity to promote a therapy to a patient audience, to direct a meeting agenda, to guide content of educational materials, to promote participation in a specific clinical trial, to influence the outcome of a specific research program, or to provide exclusive support of a particular research program.

3.4. It is ideal that any financial contribution to the patient advocacy organization be made either as (1) unrestricted funding or (2) sponsorship of a specific activity initiated by the patient advocacy organization to support its stated mission.

3.5. All donations must be given in a named manner (i.e., not given anonymously). The patient advocacy organization is transparent and open about its funding sources. Any funding provided by a biopharmaceutical company is disclosed by the patient organization (e.g., “project supported by…”).

3.6. The patient advocacy organization seeks donations in a fair and transparent manner among multiple partners to avoid real or perceived exclusive relationships and to maintain autonomy. Relying on a single partner and/or industry can compromise sustainability and autonomy; therefore, the organization attempts to receive donations from more than one partner and/or industry whenever possible.

3.7. The patient advocacy organization establishes metrics to evaluate the effectiveness of an activity or initiative in which it has collaborated with a biopharmaceutical company and regularly communicates back to the company results of the specific project or use of funds.

3.8. The patient advocacy organization may provide consultation to a biopharmaceutical company if the consultation is consistent with the mission of the organization and allows it to maintain autonomy. Terms of these services will be documented by mutual agreement between the patient organization and the biopharmaceutical company. The leaders (i.e., staff, board members, committee members) of a patient advocacy organization will not operate as independent consultants to a biopharmaceutical company outside of their roles within the patient organization.

3.9. The leaders of the patient advocacy organization will not accept personal honoraria to speak on behalf of the organization but, alternatively, may have the honoraria given to the organization.

3.10. Travel expenses incurred to participate in advisory board meetings or disease awareness activities may be reimbursed directly to the individual patient or to the patient advocacy organization.

3.11. Any transfers of value or benefits provided to the patient advocacy organization by a biopharmaceutical company should be documented by a signed agreement between the two organizations.

**4. Clinical Trial Communication and Support**

As a representative of the patient community, the patient advocacy organization is committed to providing education and resources about clinical trials to its members. The organization informs the patient community about open and upcoming clinical trials. The organization also educates patients about their vital role during the clinical trial process, from design to conclusion. Overall community participation in clinical trials is essential to advance the science and understanding of the disease.

4.1. The patient advocacy organization acts as a conduit for information about clinical trials by providing education and resources to the patient community.

4.2. The choice to participate in any particular trial is an individual one; the patient advocacy organization does not seek to influence that choice, but rather, assists patients and families in making informed decisions through education and awareness.

4.3. The patient advocacy organization disseminates accurate and fair-balanced information about clinical trials without adding commentary or opinion that may influence an individual’s decision in any way.

4.4. To support optimal clinical trial design and communication, the patient advocacy organization may provide the biopharmaceutical company with community-wide observations, needs, and barriers to participation.

4.5. The patient advocacy organization shall develop and communicate a position on their role in the sharing of individual clinical trial experiences in social media. Disclosing clinical trial experiences in social media can compromise the validity and conduct of a clinical trial and has implications for individual health privacy. However, ultimately, the choice to share information is personal; thus, a patient organization cannot dictate what information clinical trial participants do or do not share in public forums. The patient organization may provide the community with educational materials on the potential implications, both positive and negative, of disclosing clinical trial experiences publicly.

4.6. Board and committee members of the patient advocacy organization have a responsibility to represent the patient organization in their conduct. Information about clinical trials that is accessible to the community through social media, including in personal blogs or other forms of communication, should adhere to the principles outlined in these Guidelines.

4.7. At the end of a clinical trial, the patient advocacy organization asks the biopharmaceutical company to provide a summary of available trial results for trial participants and the patient community in a timely fashion. The patient advocacy organization requests that the company inform patients, in a way that is easily understandable and offers the option to seek clarification, about the ways in which the patients’ participation has resulted in a valuable contribution to the knowledge base or to the development of a therapy.

## Abbreviations

CTTI: Clinical Trials Transformation Initiative
EFPIA: European Federation of Pharmaceutical Industries and Associations (Europe)
EMA: European Medicines Agency
FDA: Food and Drug Administration
IFOPA: International Fibrodysplasia Ossificans Progressiva Association
PCWP: Patients’ and Consumers’ Working Party
PhRMA: Pharmaceutical Manufacturers of America
